# Supplementary material for: R‐loop formation during S phase is restricted by PrimPol‐mediated repriming
Source: EMBO J. 2018 Nov 26;38(3):e99793. doi: 10.15252/embj.201899793 (PMC6356060; doi:10.15252/embj.201899793)
Supplement: Supplementary file 1 — Appendix [file EMBJ-38-e99793-s001.pdf]

Appendix for:

S phase R-loop formation is restricted by PrimPol-mediated repriming

Saša Šviković[1], Alastair Crisp[1], Sue Mei Tan-Wong[2], Thomas A. Guilliam[3], Aidan J. Doherty[3], Nicholas J. Proudfoot[2], Guillaume Guilbaud[1] and Julian E. Sale[1,*].

[1] MRC Laboratory of Molecular Biology, Francis Crick Avenue, Cambridge, CB2 0QH, UK
[2] Sir William Dunn School of Pathology, South Parks Road, Oxford, OX1 3RE, UK
[3] Genome Damage & Stability Centre, School of Life Sciences, University of Sussex, Brighton, BN1 9RQ, UK
[*] to whom correspondence should be addressed: jes@mrc-lmb.cam.ac.uk

Table of Contents

Appendix Figure S1. Fluctuation analysis for the generation of Bu-1a loss variants in *rev1* cells harbouring $(GAA)_{10}$ or $(GAA)_{20}$ in place of the +3.5 G4 in *BU-1A*.

Appendix Figure S2. The S9.6 R-loop signal in *BU-1* of *primpol*$(GAA)_{10}$ is sensitive to pretreatment with RNase H, but not RNase III.

Appendix Figure S3. R-loop formation across *BU-1* in WT ($BU\text{-}1^{GAA10}$) cells.

Appendix Figure S4. Potential G4 and H-DNA forming sequences in the TTS of *BU-1*.

Appendix Figure S5. Ectopic expression of YFP-GgRNaseH1.

Appendix Figure S6. Expression of an RNaseH1 hybrid binding domain fused to mCherry in $BU\text{-}1^{(GAA)10}$ DT40 cells.

Appendix Figure S7. DRIP-qRT-PCR at four loci through the cell cycle.

Appendix Figure S8. R-loop peak widths in wild type and *primpol* cells.

Appendix Figure S9. Number of peaks overlapping a gene as a function of the distance allowed for an overlap.

Appendix Figure S10. Length distribution of H-DNA and G4 motifs in the chicken and human genomes.

Appendix Table: Oligonucleotides

Appendix Figure S1

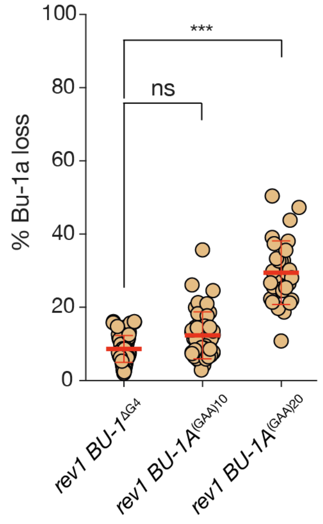

Fluctuation analysis for the generation of Bu-1a loss variants in *rev1* cells harbouring (GAA)$_{10}$ or (GAA)$_{20}$ in place of the +3.5 G4 in *BU-1A*. In all cases the +3.5 G4 on the *BU-1B* has been deleted. Statistical differences calculated using one-way ANOVA. For all panels, mean and 1 SD reported. *** $p \leq 0.001$, ns = not significant).

Appendix Figure S2

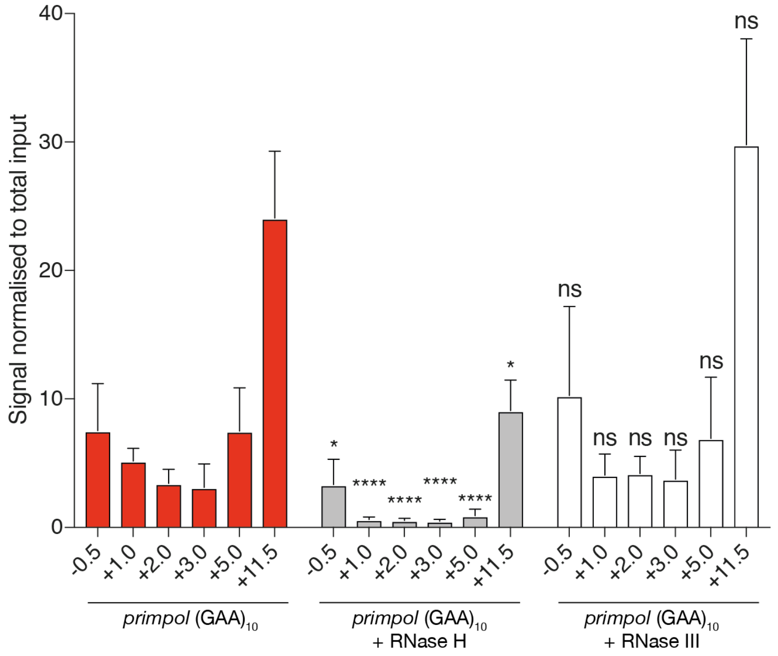

The S9.6 R-loop signal in *BU-1* of *primpol*(GAA)$_{10}$ is sensitive to pretreatment with RNase H, but not RNase III. All results presented as mean with 1SD. Statistical differences calculated using an unpaired t-test. For all panels, the mean and 1 SD are reported. (* $p \leq 0.05$ **** $p \leq 0.0001$, ns = not significant (unpaired t-test), compared to untreated *primpol*(GAA)$_{10}$).

Appendix Figure S3

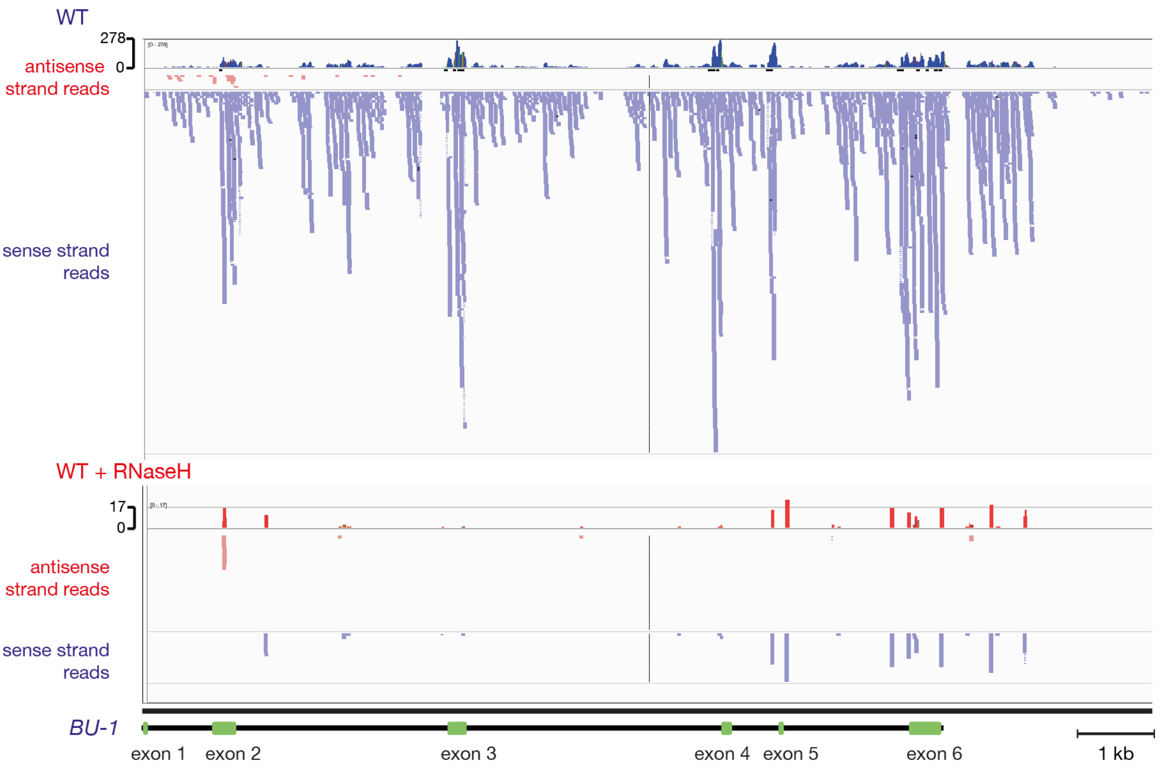

R-loop formation across *BU-1* in WT (BU-1^{GAA10}) cells. RNA-DIP reads mapping to the *BU-1* locus separated by origin into antisense (red) and sense (blue). The lower panel shows reads mapping after pre-treatment with RNase H.

Appendix Figure S4

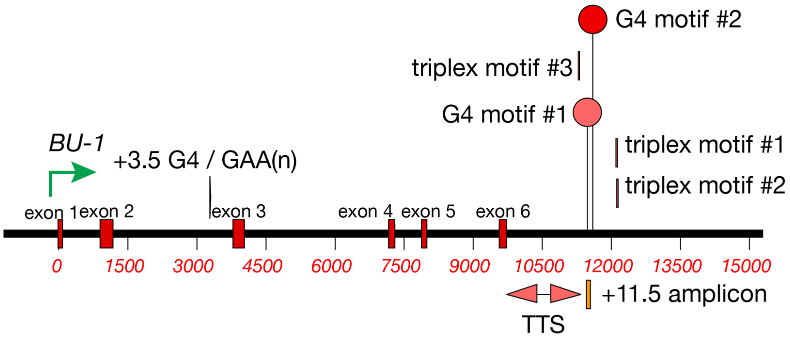

Potential G4 and H-DNA forming sequences in the TTS of *BU-1*. The region around the +11.5 position of *BU-1* contains a number of leading strand structure-forming motifs, which may account for the increase in the R-loop signal in this region in *primpol* cells. The sequences indicated on the diagram are as follows:

G4 motif #1: 5′-GGGTGGGCTGGGGAACAGAGGTCATGAGGG

G4 motif #2: 5′-GTGGCCAGAAGGGAGAGGGAGGGG

Triplex motif #1: 5′-TTTATAGTTAATGCTGGAAAAAAGAAAAAA

Triplex motif #2: 5′-GAAAAAAGAAAAAAGTTGAAAAAGAAAAACA

Triplex motif #3: 5′-GGTTTTTTTTTTCCATTTTCCCTGCA

Appendix Figure S5

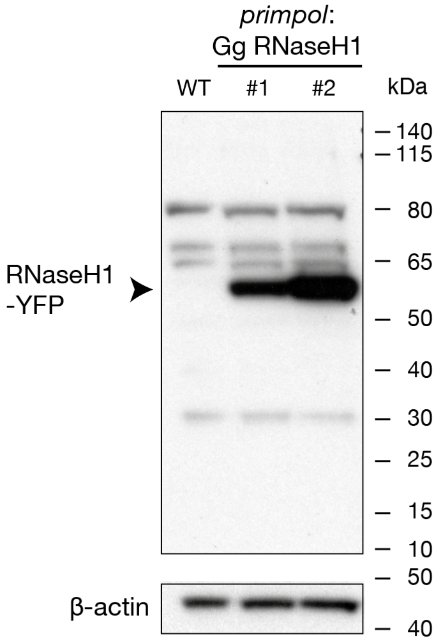

Ectopic expression of YFP-GgRNaseH1. Anti-YFP western blot showing two representative chicken RNaseH1-expressing clones.

Appendix Figure S6

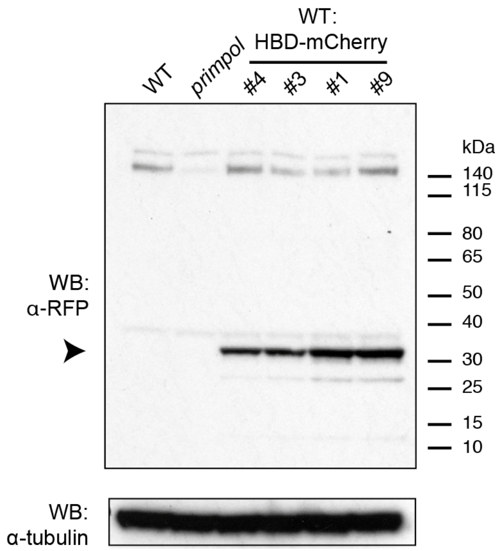

Expression of an RNaseH1 hybrid binding domain fused to mCherry in *BU-1*$^{(GAA)10}$ DT40 cells. The fluctuation analysis data from four clones shown here were pooled to generate the graph shown in Fig 3E.

Appendix Figure S7

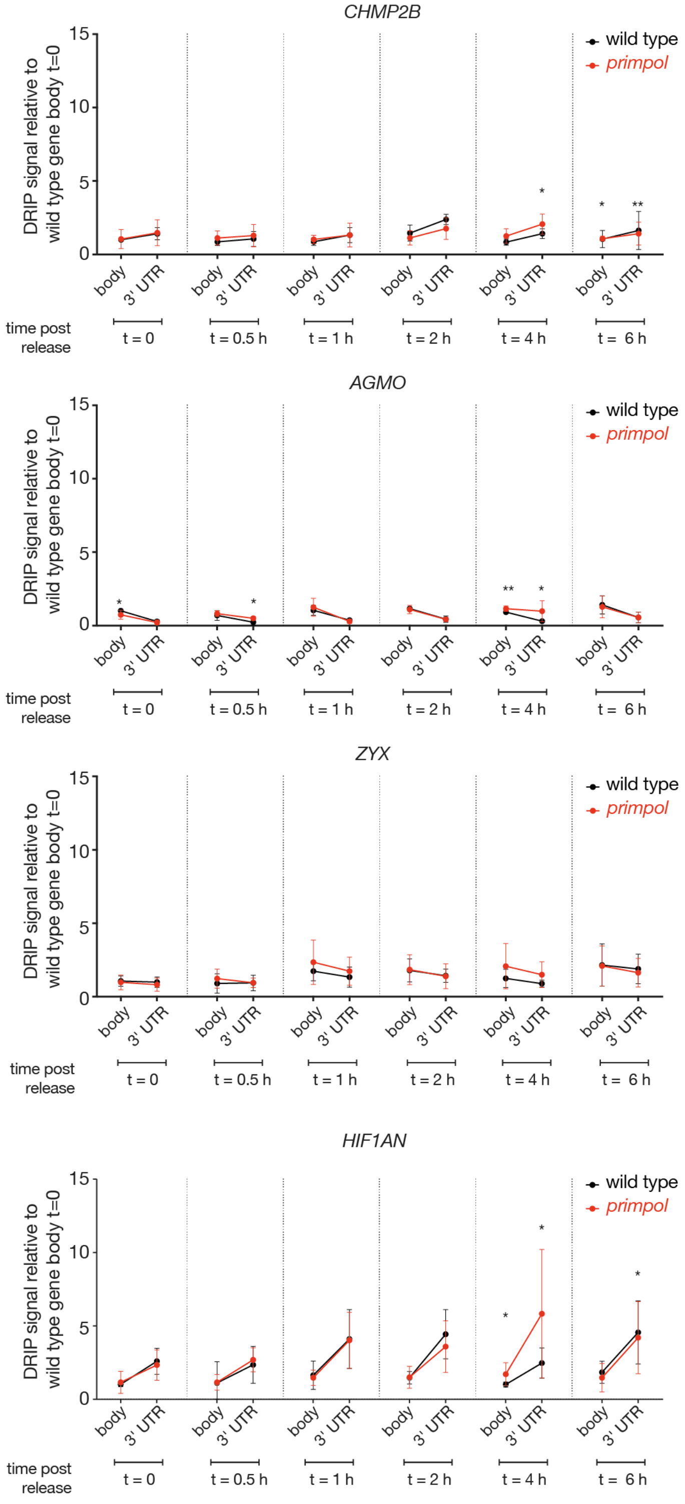

DRIP-qRT-PCR at four loci through the cell cycle. For each gene two sets of primers were used to interrogate the precipitated DNA, one in the gene body and one at the TSS. *CHMP2B, AGMO* and *ZYX* do not have identifiable secondary structures within 5kb of either primer pair. In *HIFAN,* a G4 motif is located c. 1000 bp 5’ of the 3’ UTR primer pair. Error bars 1 SD of 4 biological replicates. * $p \leq 0.05$; unpaired t-test

Appendix Figure S8

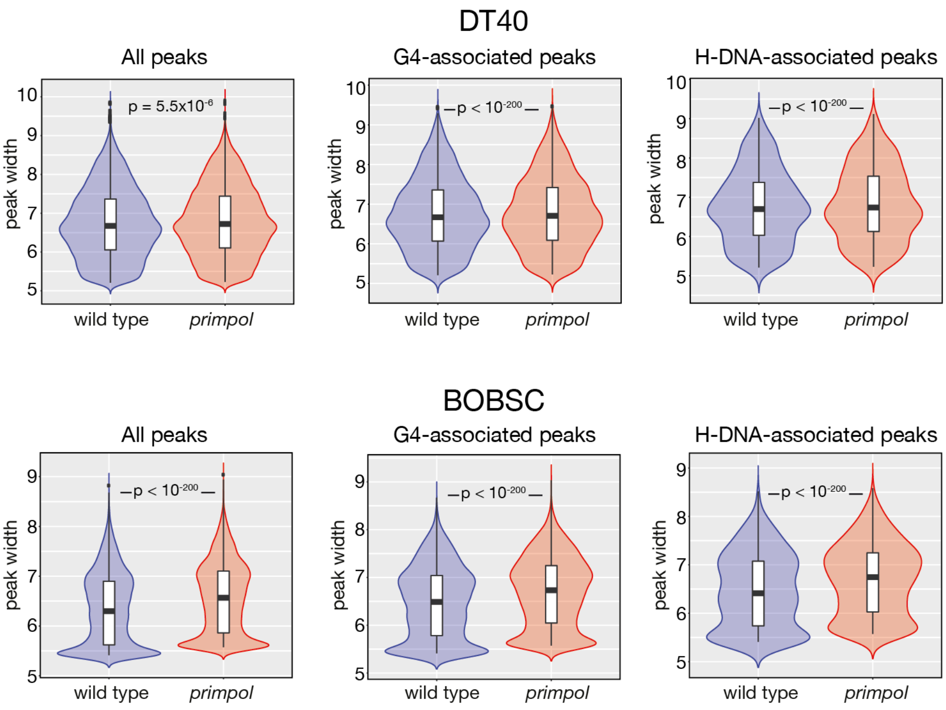

R-loop peak widths in wild type and *primpol* cells. Top row: DT40 DRIP peaks. Bottom row: BOBSC RNA-DIP peaks. P values calculated with Mann-Whitney U test.

Appendix Figure S9

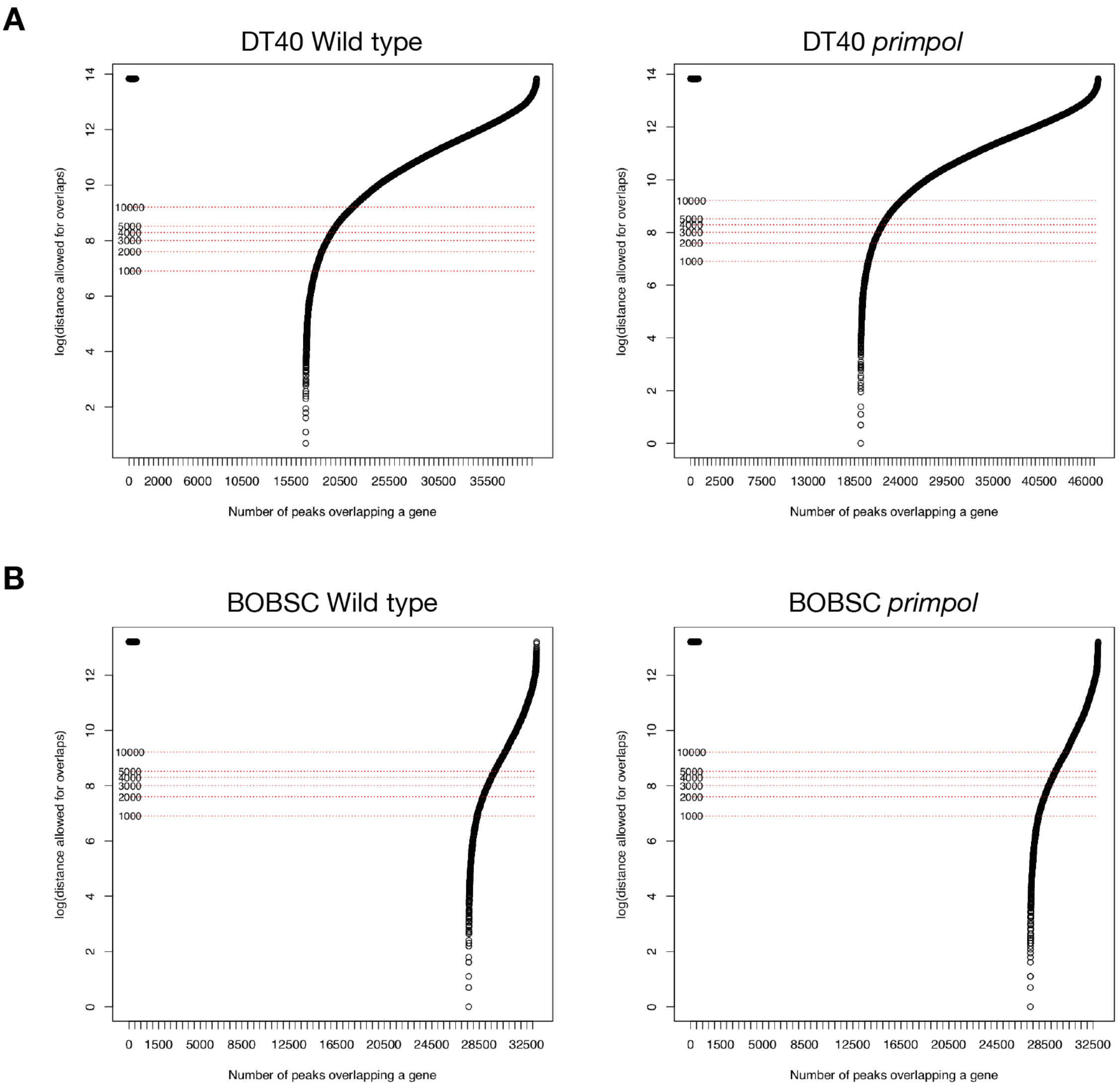

Number of peaks overlapping a gene as a function of the distance allowed for an overlap. In both the A) chicken DRIP and B) RNA-DIP datasets the number of peaks identified asymptotes at approximately 1000 bp from the gene. Thus, our threshold of 1bp or more overlap with between a peak and the gene will be robust to the very small change in peak width between the wild type and *primpol* datasets.

Appendix Figure S10

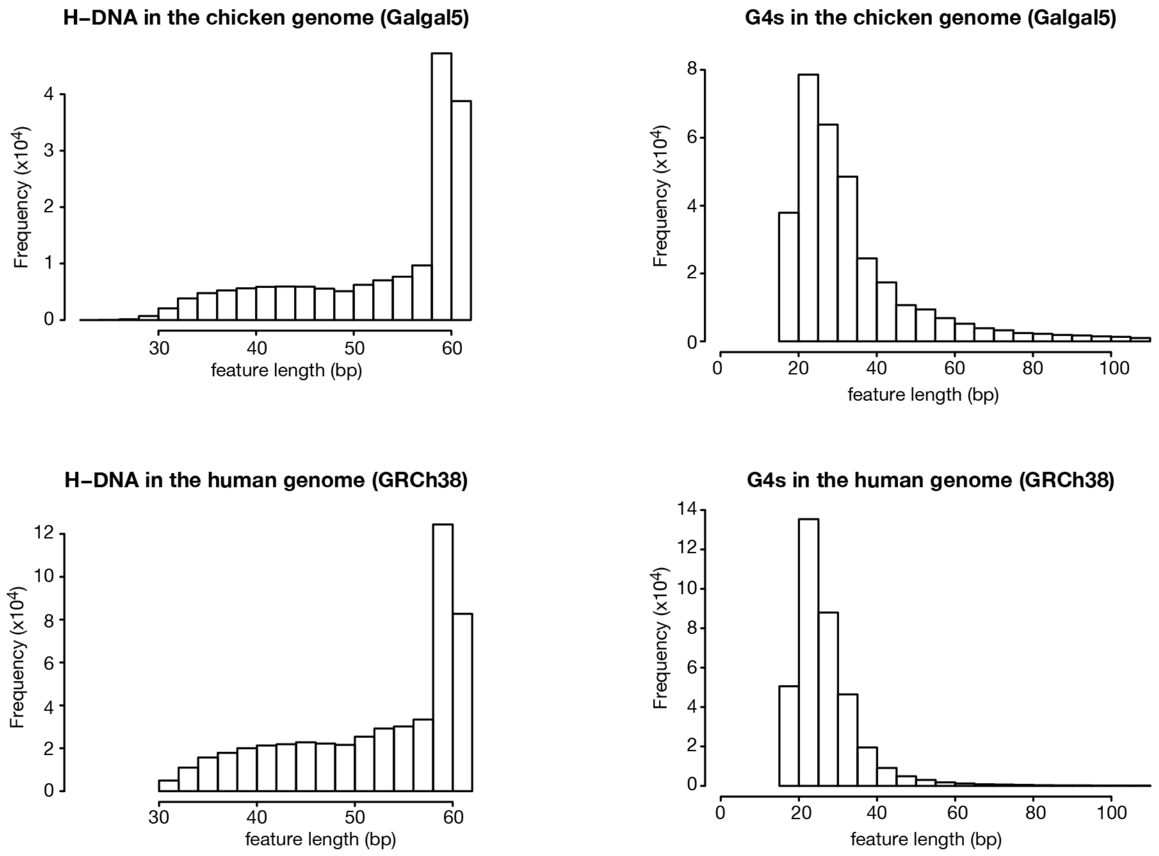

Length distribution of H-DNA and G4 motifs in the chicken and human genomes. Total number of motifs identified: Galgal5: H-DNA 167377; G4 335587. GRCh38: H-DNA 504650; G4 364321.

Appendix Table: Oligonucleotides.

Oligonucleotides used for TNR cloning and BU-1 genotyping

Oligonucleotide name	Sequence (5’-3’)
(GAA)10	CGCGTGAAGAAGAAGAAGAAGAAGAAGAAGAAGAA
(GAA)20	CGCGTGAAGAAGAAGAAGAAGAAGAAGAAGAAGAAGAAGAAGAA<br>GAAGAAGAAGAAGAAGAAGAA
(GAA)30	CGCGTGAAGAAGAAGAAGAAGAAGAAGAAGAAGAAGAAGAAGAA<br>GAAGAAGAAGAAGAAGAAGAAGAAGAAGAAGAAGAAGAAGAAGA<br>AGAAGAA
(TTC)10	CGCGTTTCTTCTTCTTCTTCTTCTTCTTCTTCTTC
(TTC)20	CGCGTTTCTTCTTCTTCTTCTTCTTCTTCTTCTTCTTCTTCTTC<br>TTCTTCTTCTTCTTCTTCTTC
(TTC)30	CGCGTTTCTTCTTCTTCTTCTTCTTCTTCTTCTTCTTCTTCTTC<br>TTCTTCTTCTTCTTCTTCTTCTTCTTCTTCTTCTTCTTCTTCTT<br>CTTCTTC
GAA20 MluI-BbsI-BsmBI-NcoI-Mlu	CGCGTTTTGAAGACTTGAAGAAGAAGAAGAAGAAGAAGAAGAAG<br>AAGAAGAAGAAGAAGAAGAAGAAGAAGAAGAAGAGAGACGT<br>TTCCATGGTTTA
TTC20 MluI-BbsI-BsmBI-NcoI-Mlu	CGCGTAAACCATGGAAACGTCTCTCTTCTTCTTCTTCTTCTTCT<br>TCTTCTTCTTCTTCTTCTTCTTCTTCTTCTTCTTCTTCTTCTTC<br>AAGTCTTCAAAA
GAA22 MluI-BbsI-BsmBI-NcoI-Mlu	CGCGTTTTGAAGACTTGAAGAAGAAGAAGAAGAAGAAGAAGAAG<br>AAGAAGAAGAAGAAGAAGAAGAAGAAGAAGAAGAAGAAGAAGAG<br>AGACGTTTCCATGGTTTA
TTC22 MluI-BbsI-BsmBI-NcoI-Mlu	CGCGTAAACCATGGAAACGTCTCTCTTCTTCTTCTTCTTCTTCT<br>TCTTCTTCTTCTTCTTCTTCTTCTTCTTCTTCTTCTTCTTCTTC<br>TTCTTCAAGTCTTCAAAA
BU-1 [6-FAM] F	[6FAM]ATTCATTATATAGGAAGCCTCTGGC
BU-1 [HEX] R	[HEX]TGATGAGCAGACTCAGCAGTAA

Oligonucleotides used for generating and genotyping *primpol* human cell mutants with CRISPR/Cas9

Oligonucleotide name	Sequence (5’-3’)
PRIMPOL gRNA 1 F	ACCGAGCTTGCACACAGCATTTTC
PRIMPOL gRNA 1 R	AAACGAAAATGCTGTGTGCAAGCT
PRIMPOL gRNA 2 F	ACCGTTTAACAAACCTGCCAACCC
PRIMPOL gRNA 2 R	AAACGGGTTGGCAGGTTTGTTAAA
PRIMPOL 5’HA F2	TAGCACTCAGTGGGATCGATGTCGACCACTACCTGTACCACCTGATG
PRIMPOL 5’HA R2	GCTATTTAATTAATTGCATAGAATTGGGTTTCCATCAAGCAAAATGC
PRIMPOL 3’HA F1	GTAAATGCGGCCGCTAGGCATCCATGGCTGACCTTTGGTAAAACCAG<br>GC
PRIMPOL 3’HA R1	TAAAACGACGGCCAGTGGCATTGATAGGGGTGGTGGGGGACTTTAT

Oligonucleotides used for bisulphite sequencing

Oligonucleotide name	Sequence (5'-3')
BU-1 Meth +0.5 F SacI	GTTTCTTGAGCTCGAATATAAGGTTTTGGTATGTAGAATGT
BU-1 Meth +0.5 R NotI	GTTTCTTGCGGCCGCCTCCCTAATCACTAAAATTATATACAAAA

Primers for transgene overexpression

Oligonucleotide name	Sequence (5'-3')	Comments
RNase H1 dMLS HindIII Fw2	CTTAAAGCTTATGTTCTACGCGGTGCGCAAG	Forward primer containing HindIII restriction site used to amplify Gg RNaseH1ΔMLS
RNase H1 SalI Rev1	AGAGTCGACATGCAACTTTTGTTTACTGGCGC	Reverse primer containing SalI restriction site used to amplify Gg RNaseH1ΔMLS
EYFP SalI Fw1	ATATAGTCGACGTGAGCAAGGGCGAGGAGCTG	Forward primer containing SalI restriction site used to amplify YFP as a C-terminal tag
EYFP NotI Rev1	ATTGTTAGCGGCCGCTCATTACTTGTACAGCTCGTCCA	Reverse primer containing NotI restriction site used to amplify YFP as a C-terminal tag, with stop codon added at the end of the ORF
YFP C-term (-stop) BamHI R	TATTTCTATTGGGGGATCCCTTGTACAGCTCGTCCATGCCGAGAG	Reverse primer containing BamHI restriction site used to amplify YFP tag as an internal tag
hCdt1 C-term (-ATG) BamHI F1	CTGTACAAGGGATCCCCCAGCCCCGCCAGGCCCGCACTCC	Forward primer containing BamHI restriction site used to amplify hCdt1 [30-120] fragment as a C-terminal tag
hCdt1 C-term (+TAA) NotI R1	TATATCTAGTTAGCGGCCGCTCATTAGATGGTGTCCTGGTCCTGC	Reverse primer containing NotI restriction site used to amplify hCdt1 [30-120] fragment as a C-terminal tag, with stop codon added at the end of the ORF
hGeminin [1-110] BamHI F	TATACTCTAGGATCCATGAATCCCAGTATGAAGCAGAAACAAG	Forward primer containing BamHI restriction site used to amplify hGeminin [1-110] fragment as a C-terminal tag
hGeminin [1-110] (+TAA) NotI R	CTATTGTTTAGCGGCCGCTCATTACAGCGCCTTTCTCCGTTTTTCTGCC	Reverse primer containing NotI restriction site used to amplify hGeminin [1-110] fragment as a C-terminal tag, with stop codon added at the end of the ORF
YFP N-term tag HindIII F	ATATTATCAAAGCTTATGGTGAGCAAGGGCGAGGAGCTG	Forward primer containing HindIII restriction site used to amplify YFP as a N-terminal tag, with a start codon added at the beginning of ORF
YFP N-term tag Sal R	ATAACGTAGTCGACCTTGTACAGCTCGTCCA	Reverse primer containing SalI restriction site used to amplify YFP as a N-terminal tag

Hs PrimPol (-ATG) SalI F	TTATTCTATAGTCGACAATAG AAAATGGGAAGCAAAAC	Forward primer containing SalI restriction site used to amplify hPrimPol, with the start codon omitted
HsPrimPol FL (+TAA) NotI R	ACGATATAATAATGCGGCCGC TTATTACTCTTGTAATACTTC TATAAT TAGTTCATCAGGAATTTC	Reverse primer containing NotI restriction site used to amplify full length hPrimPol, with the stop codon added at the end of the ORF
HsPrimPol 1-546 (+TAA) NotI R	ACGATATAATAATGCGGCCGC TTATTACACTTCACTGTTATA ACTGAG AAGA	Reverse primer containing NotI restriction site used to amplify hPrimPol ΔRBM-B cDNA, with the stop codon added at the end of the ORF
Hs HBD HindIII F	GAAAAGCTTATGTTCTATGCC GTGAGG	Forward primer containing HindIII restriction site used to amplify HBD-mCherry
mCherry (+stop codon) NotI R	TTATGCGGCCGCTCACTTGTA CAGCTCGTCCATGCCGCCGG	Reverse primer containing SalI restriction site used to amplify HBD-mCherry, with a stop codon added at the end of the ORF

ChIP and DRIP qPCR primers

Oligonucleotide name	Sequence (5'-3')
BU-1 -0.5 F	AAGAGCCTTGCGAAGTTCAG
BU-1 -0.5 R	TTTTCTGCAGTGCCAATGAC
BU-1 +1.0 F	GGCAGCTCAGCAAAGTTTCC
BU-1 +1.0 R	GACCACAGCCGTGGAACAGTTA
BU-1 +2.0 F	ACTGTTCACCCACTGAAGTTGAAA
BU-1 +2.0 R	GGTTTGTTTAGCAAAAGGGAGTAG
BU-1 +3.0 F	GCAGTGTGGCAGATGTAGGA
BU-1 +3.0 R	GTAGGTGTCAACGCCAGACA
BU-1 +5.0 F	GGAACAAGAAGGCAAACAGC
BU-1 +5.0 R	GGCAGCTACCCAGAACAAAC
BU-1 +11.5 F	TACTGTTGGCTACAGCTGGC
BU-1 +11.5 R	TCCTACAGCCTCTCCTGGTC
ρ-globin 8.99 F	GGGCCCAATGAACCAGAAA
ρ -globin 8.99 R	TGTTCCCCAGCAACGCA
ρ -globin 21.37 F	CTCTGTGCTCAGCATCCTTCAAT
ρ -globin 21.37 R	CCTTTCGGCACTTTCTTCCTTT
Gg GAPDH promoter F	TTGCCGAGCAAACAGAGG
Gg GAPDH promoter R	CCCCATCTTGAGGTTACGAC
Gg GAPDH +1.0 F	TGTTTGTGATGGGTGTCAAC
Gg GAPDH +1.0 R	GCATTGCTGGGAAAGAAAGAAG
RPLP0 (upstream) F	CAATGGGCTGCGCGAGAG
RPLP0 (upstream) R	GAGGGCTCCGGGAAAGAAG
RPLP0 (2nd exon) F	TGTTTCGTGGTGGGAGCG
RPLP0 (2nd exon) R	GCATCATCGTGTTCTTCCCC
ch28S rDNA 2.1 F	GCAGGTCTCCAAGGTGAACA
ch28S rDNA 2.1 R	CCAGCCCTTAGAGCCAATCC

AGMO gene F	CTGAAGATGTGGCTGCAAAA
AGMO gene R	CAGGCTCCTCTGCTGCTAAT
AGMO 3'UTR F	TTTTTGCATCCAAGGTGTCA
AGMO 3'UTR R	TGCTTTATACCGGATTGCTACA
CHMP2B gene F	GTGGTGCAGCTTAATGCAGA
CHMP2B gene R	GGGTTCGTATCTGCCAACAT
CHMP2B 3'UTR F	GCTGGCGGTGTTATCTCCTA
CHMP2B 3'UTR R	TCGTTTCTGCCAAGATGACA
HIF1AN gene F	TGGCTTTAAGGCAGCTTGAT
HIF1AN gene R	CTCGCAAAAATGCTCACAAA
HIF1AN 3'UTR F	TGCCCTTTTTACCAAACCTG
HIF1AN 3'UTR R	AGGAAGGGAACACCACACTG
ZYX gene F	TGTCCTGTTTGTCCTCACCA
ZYX gene R	CCATGAGGAAAGAGGGATCA
ZYX 3'UTR F	TCAGGGTGGGCAAGAATAAC
ZYX 3'UTR R	GAAAACCGAGAGCTCCACAG
