# Supplementary material for: R‐loop formation during S phase is restricted by PrimPol‐mediated repriming
Source: EMBO J. 2018 Nov 26;38(3):e99793. doi: 10.15252/embj.201899793 (PMC6356060; doi:10.15252/embj.201899793)
Supplement: Supplementary file 2 — Expanded View Figures PDF [file EMBJ-38-e99793-s002.pdf]

Expanded View Figures

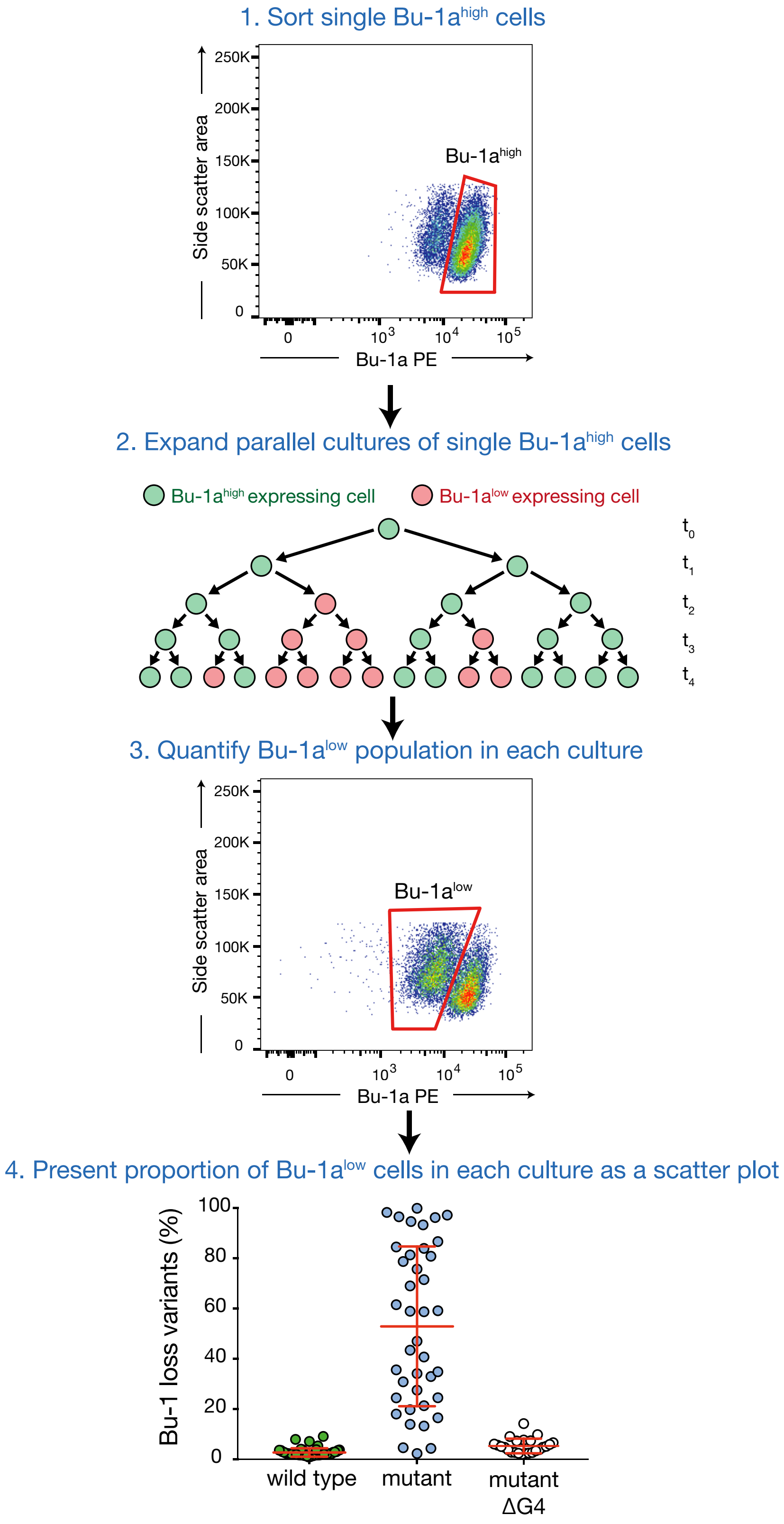

Figure EV1. Outline of the *BU-1* fluctuation analysis (adapted from Schiavone *et al*, 2014).

Single Bu-1a^{high} cells are sorted into 96-well plates. As these clones expand, stochastic switching of the expression state of *BU-1* to a lower level occurs. The switch from Bu-1a^{high} to a lower expression state is irreversible. After 17–20 days in culture, the proportion of Bu-1a^{low} cells (or Bu-1a loss variants) in each population is determined and plotted. The median percentage of loss variants generated correlates with the per-division probability of expression state switching (Schiavone *et al*, 2014).

© 2018 MRC Laboratory of Molecular Biology

Figure EV2. Strategy for cloning uninterrupted GAA tracts.

A The structure of MluI-BbsI-(GAA)n-BsmBI-NcoI-MluI linker.

B Iterative elongation of a GAA tract by coordinated restriction with type IIS and type IIR restriction enzymes (Scior *et al*, 2011). Once ligated, the uninterrupted GAA tract can be excised from the vector and subcloned into targeting constructs with compatible overhangs.

C Electropherogram of Sanger sequencing reactions of plasmids bearing $(GAA)_{30}$, $(GAA)_{50}$ and $(GAA)_{75}$ repeats showing uninterrupted tracts. Green peaks correspond to adenine and black to guanine.

© 2018 MRC Laboratory of Molecular Biology

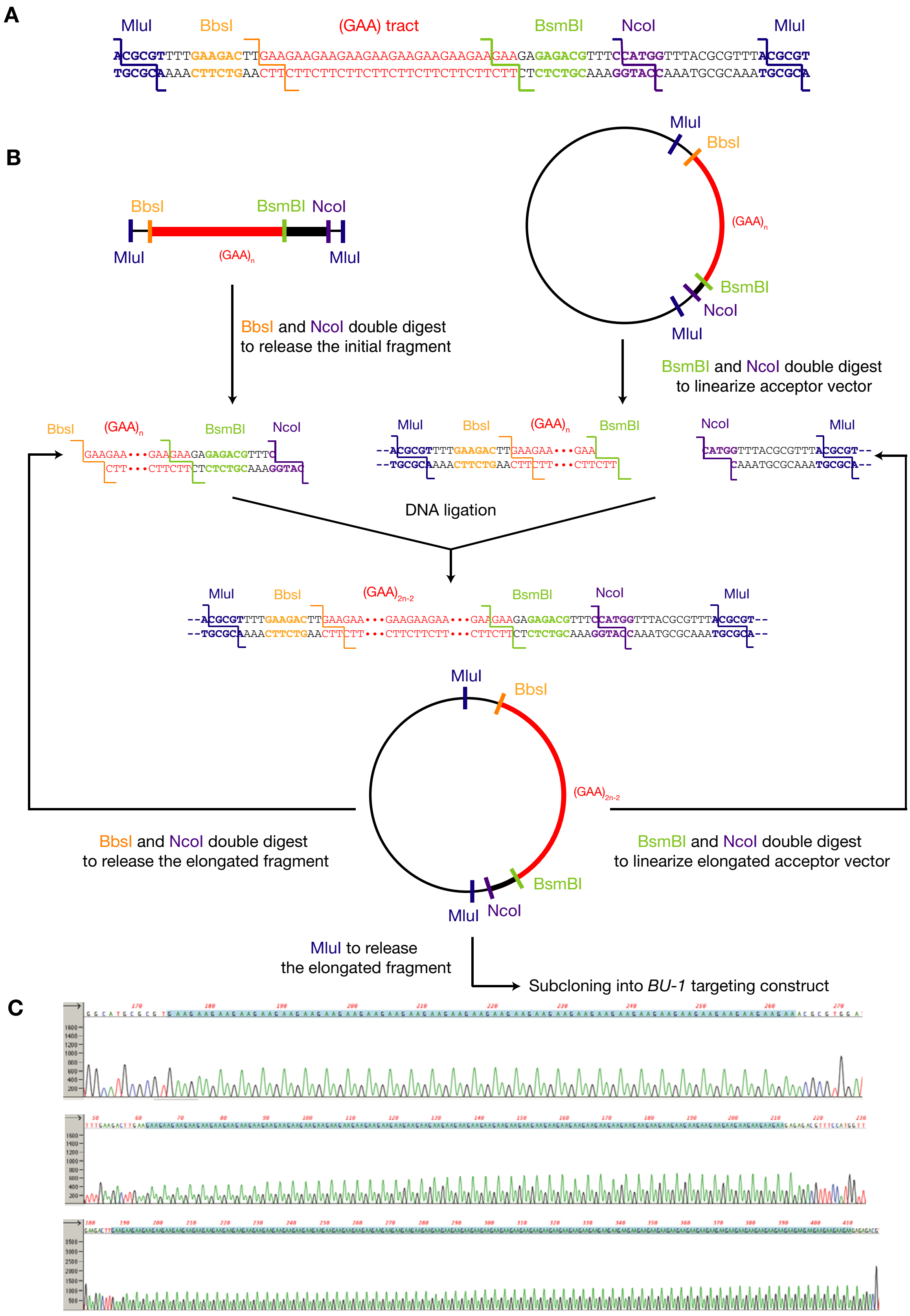

Figure EV2.

© 2018 MRC Laboratory of Molecular Biology

Figure EV3. Transcriptional and epigenetic changes in *BU-1* harbouring $(GAA)_n$ repeats.

A Chromatin-associated RNA at *BU-1* increases as a function of +3.5 $(GAA)_n$ repeat length. The coloured groups of bars represent results from cells with different repeat lengths. The *X*-axis shows the position of the primer pairs used to interrogate the cDNA made from the chromatin-extracted RNA. Mean ± SD of three biological replicates reported. Test for significance at +3.0 (the primer pair closest to the engineered $(GAA)_{10}$ repeat) performed with the two-tailed *t*-test (ns = not significant; $^*P \leq 0.05$; $^{****}P \leq 0.0001$).

B FACS plot showing *primpol* $(GAA)_{10}$ populations expressing Bu-1a at high, medium and low states, and their relation to wild-type cells.

C ChIP analysis of H3K4me3 across *BU-1* locus. The right panel depicts enrichment of H3K4me3 signal at the *GAPDH* and β-globin loci as a positive and negative control, respectively.

D ChIP enrichment of H3K36me3 across *BU-1*. The right panel shows H3K36me3 enrichment at the *RPLP0* and β-globin control loci.

E Representative plots of the methylation status of CpG dimers, shown as circles. For clarity, 10 random sequences are depicted. Filled circles represent methylated and open circle unmethylated CpGs. On the right: bar chart depicting methylation in different expression status. *n* represents the number of analysed molecules.

F Fragment length analysis assay for genetic instability in *BU-1A* locus. Top panel: map of *BU-1* +3.5 kb site with primers used labelled with 6-FAM and HEX. ΔG4 allele is amplified as a 126-bp product; knock-in of $(GAA)_{10}$ with MluI overhangs introduces an additional 35 bp, producing a 161-bp product. Lower panel: overlay of 24 electropherograms obtained from *primpol* $BU\text{-}1A^{(GAA)10}$ clones retrieved following fluctuation analysis. Two peaks (blue and green) at each position of *X*-axis correspond to forward and reverse strands labelled with different fluorophores. The trailing edge behind major peak is an artefact of PCR amplification of repetitive DNA called stutter peaks. Although stutters could be the same size as expected mutagenic events, they do not present more than 10% of the amount of the major PCR product and are always shorter. These two features permit distinction between artefacts and genetically unstable alleles (Lee *et al*, 2010).

Data information: In (C and D), modified H3 signal is normalised to total H3. Each bar reports 2–4 biological replicates with standard error of mean. IgG background is only depicted at control loci as grey points, as the specific H3K4me3 or H3K36me3 signals were 100- to 1,000-fold higher than the IgG control. Statistical comparison between samples: unpaired two-tailed *t*-test (ns = not significant; $^*P \leq 0.05$; $^{***}P \leq 0.001$; $^{****}P \leq 0.0001$).

© 2018 MRC Laboratory of Molecular Biology

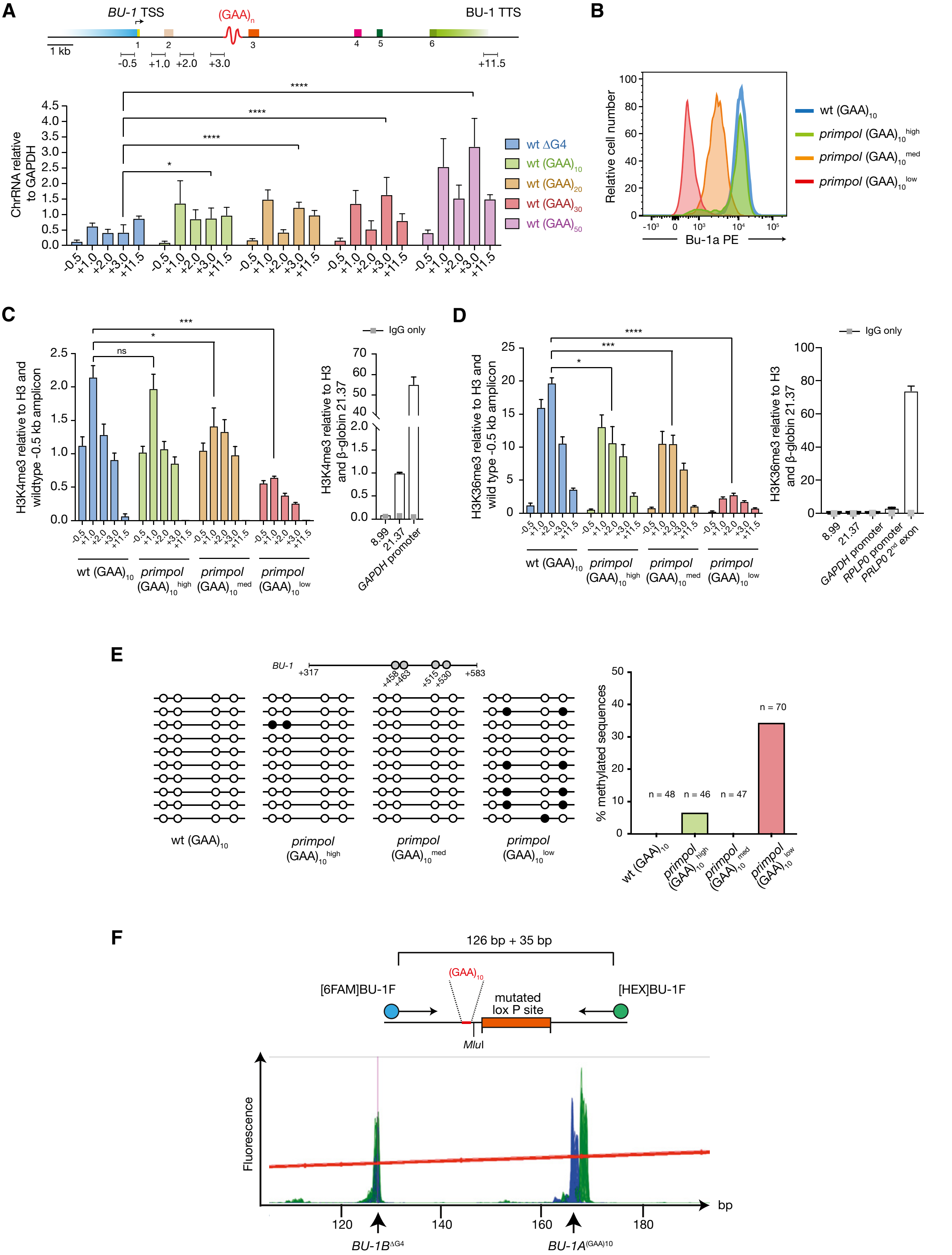

Figure EV3.

© 2018 MRC Laboratory of Molecular Biology

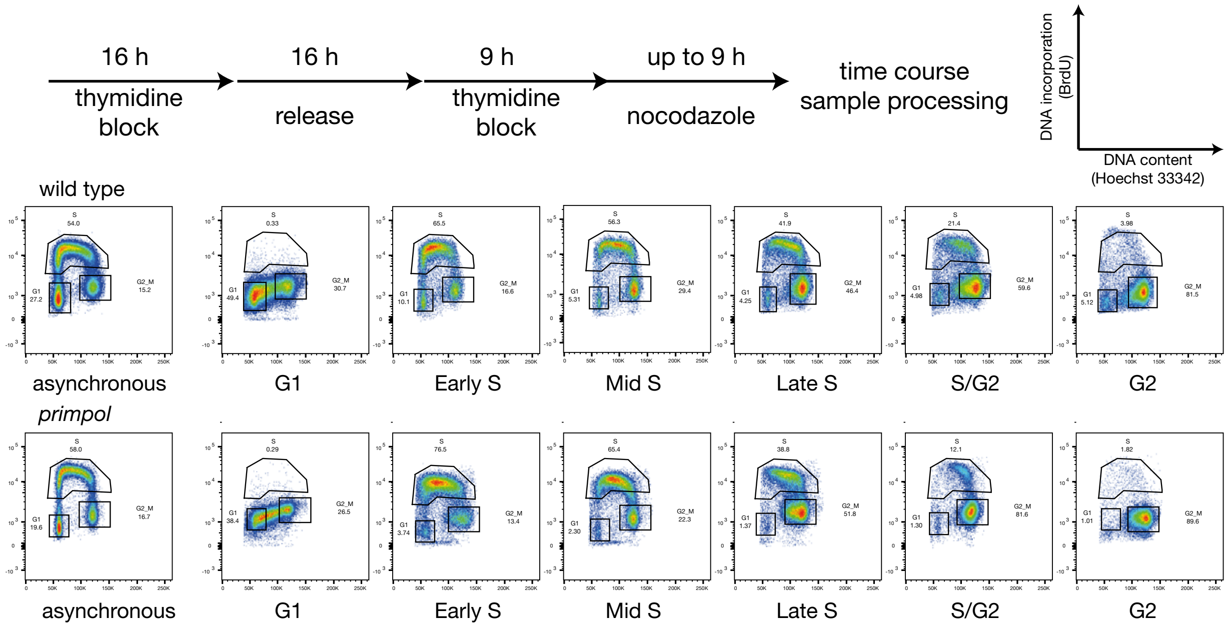

Figure EV4. Synchronisation of DT40.

Cell cycle synchronisation and capture of cells progressing through different phases of the S phase. Top panel shows the synchronisation scheme with double thymidine block and release in nocodazole. FACS plots depict 2D cell cycle analysis of synchronised cells at different stages after release, revealed by double staining for BrdU incorporation and DNA content (Hoechst 33342).

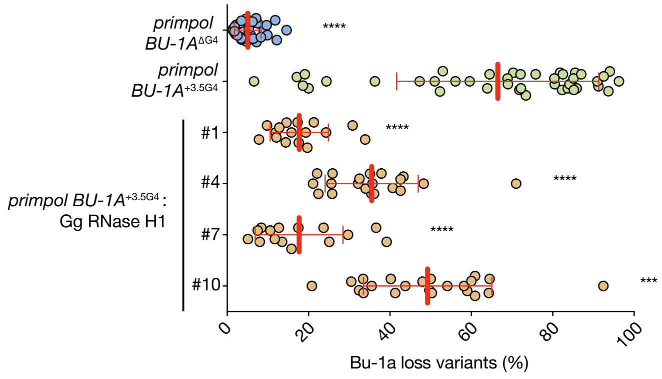

Figure EV5. Overexpression of chicken RNase H1 reduces G4-induced *BU-1* epigenetic instability in *primpol* cells.

Fluctuation analysis was performed on four *primpol BU-1A*$^{+3.5G4}$ clones. One-way ANOVA was used to calculate differences in *BU-1A* instability between *primpol BU-1A*[+3.5G4] and other cell lines, including *BU-1A*$^{\Delta G4}$ (*****P* ≤ 0.0001, ****P* ≤ 0.001). Circles represent individual clones (minimum of 18) analysed for each cell line, with mean and SD reported as line and whiskers.

© 2018 MRC Laboratory of Molecular Biology
